# The Neuroscience of Marketing: Non-invasive Neuromarketing Methods for Understanding Consumer Choice and Priorities

**DOI:** 10.12688/f1000research.168220.1

**Published:** 2025-10-17

**Authors:** Pradeep K, Sathya Prasad P.V, Rajalakshmi S A, Chriso Ricky Gill J, Jisha V. G

**Affiliations:** 1 Nitte Institute of Communication, Nitte (Deemed to be University), Mangaluru, Karnataka, 575018, India; 2 Department of Social Work, Amrita School of Social and Behavioural Sciences, Amrita Vishwa Vidyapeetham, Coimbatore, Tamilnadu, 641112, India; 3 Department of English, Vel Tech High Tech Dr. Rangarajan Dr. Sakunthala Engineering College, Avadi, India; 4 Department of English, Noorul Islam Centre for Higher Education, Kumaracoil, Thuckalay, Tamilnadu, India

**Keywords:** Neuroscience of Marketing, Neuromarketing, Consumer behaviour, Marketing, non-invasive techniques

## Abstract

Neuromarketing has emerged as a rigorous interdisciplinary paradigm for probing the latent dimensions of consumer behaviour. Through synthesizing insights from cognitive science with advanced neurophysiological methodologies, it elucidates the neural and psychological mechanisms underlying decision- making process, thereby offering a nuanced extension to contemporary marketing theory.Through non-invasive techniques such as Functional Magnetic Resonance Imaging (fMRI), Electroencephalography (EEG), Positron Emission Tomography (PET), Transcranial Magnetic Stimulation (TMS), Electromyography, biometric sensors, and implicit association tests, researchers can investigate how emotion, memory, and sensory inputs shape purchasing behaviour. As interest in the neural underpinnings of consumer choice continues to grow, neuromarketing has emerged as a dynamic and evolving field. These novel approaches navigate practical insights for tailoring marketing strategies to align with consumer preferences. Understanding consumer behaviour through these advanced, non-invasive techniques is becoming essential for organisations aiming to create impactful, evidence-based campaigns. This research reviews the most prominent non-invasive techniques and evaluates how neuromarketing frameworks can reinterpret two decades of existing literature to inform future marketing practices.

## Introduction

In the global digital era and its marketing, consumer neuroscience is highly influential and viable approach to research that is garnering increased attention among academic researchers in the fields of marketing ( Lin et al., 2018; Mohd Isa & Anuar, 2024). Consumer behaviour and decision-making processes indicates that the use of neurophysiological measurements offers objective data. The sophisticated techniques applied to marketing, neuroscience offers valuable insights into the brain’s responses to marketing stimuli, thereby enhancing our understanding of the neural mechanisms underlying purchasing decisions and emotional engagement. The integration of neuroscientific methods with marketing strategies has proven effective in uncovering both the conscious and unconscious effects of marketing stimuli on consumer behavior ( Liu et al., 2025; Clement et al., 2017; Alsharif & Khraiwish, 2024). Neuromarketing techniques reveal how emotions shape consumer perceptions, brand preferences, and purchasing decisions. Sensory stimuli sight, smell, taste, touch, and hearing evoke emotions and trigger memories, creating lasting impressions ( Cordeiro, Reis, Ferreira, & Bacalhau, 2024; Gunawan, Chen & Hsu, 2023). Conversely, it is worth noting that the integration of neuroimaging as a customary research tool or technique in most social sciences remains limited, and the field of market research has been comparatively slower in recognizing the advantages offered by this methodology.

The field of neuromarketing has experienced significant growth over the past decade; however, gaining entry into the marketing domain has proven to be a formidable challenge. This can be primarily attributed to the limited availability of adequately trained cognitive neuroscience researchers, as well as the public’s apprehension regarding the ethical ramifications of neuroimaging ( Morin, 2011; Goncalves, Hu, Aliagas & Cerdá, 2024). In the beginning, 2000s witnessed the emergence of neuromarketing as a distinct interdisciplinary field, integrating neuroscience methodologies into marketing research. During this period the pioneering firms such as Bright House and Sales Brain began contribution to neuromarketing consulting services, advocating the application of cognitive neuroscience to comprehend consumer behaviour. A seminal study by McClure et al. (2004) employed functional magnetic resonance imaging (fMRI) to investigate participants’ neural responses to Coca-Cola and Pepsi ( Pradeep et al., 2022; McClure et al., 2004). The findings revealed that when participants were unaware of the brand, their preferences correlated with movement in the ventromedial prefrontal cortex, a region associated with reward processing. Conversely, brand knowledge caused increased activation in the hippocampus and dorsolateral prefrontal cortex, these areas linked to memory and higher-order cognitive functions, suggesting that brand awareness significantly impacts consumer preferences. In the field of Marketing, marketers are increasingly enthusiastic about brain imaging due to its potential to uncover consumer preferences that are not easily articulated through direct questioning. Neuroimaging provides access to subconscious data within the consumer’s brain, offering insights that can significantly enhance product design and marketing strategies. While the initial cost of such studies may be high, the long-term benefits such as improved targeting, better product development, and potentially increased sales make the investment cost-effective ( Rawnaque, Rahman, & Anwar, 2020). Despite, this novel techniques of tracing the consumer preference, behaviour, and priority are high demanded in marketing industry.

## Understanding the consumer behaviour through neuroimaging paradigm

Currently, various sophisticated technologies are employed to map neural responses related to consumer behavior and other cognitive functions. Among these, two primary neuroimaging techniques functional magnetic resonance imaging (fMRI) and positron emission tomography (PET) are commonly used in neuroscience to visualize brain activity by assessing changes in blood flow. These methods are grounded in the neurophysiological principle that mental activity increases the demand for oxygen and glucose in activated brain regions a demand that is met through increased cerebral blood flow ( Bault & Rusconi, 2020; Genco, Pohlmann, & Steidl, 2013; Wivedi & Sharma, 2024). The fMRI detects brain activity by measuring changes in blood oxygenation levels, when neurons become active, local blood flow increases, delivering more oxygenated blood flows to the region. This influx reduces the concentration of deoxygenated haemoglobin, enhancing signal and allowing for the mapping of neural activity with high spatial resolution. The Positron emission tomography, on the other hand, involves the injection of radioactive tracers that emit positrons. These tracers such as oxygen-15 and fluorodeoxyglucose (18F-FDG), accumulate in regions of the brain exhibiting increased neural activity, which corresponds to elevated blood flow ( Rawnaque, Rahman, & Anwar, 2020; Alsharif & Mohd Isa, 2024). The resulting radiotracer emissions are detected by PET scanners to produce high-resolution images that reflect patterns of regional glucose metabolism an essential marker of brain function. Alterations in glucose metabolism often indicate underlying neurological or cognitive conditions. Rong, Lulong & Zhihua (2024) observed that medical imaging technologies are advancing rapidly, with molecular imaging emerging as a particularly promising field. The novel innovation in this area have sparked interest not only in clinical neuroscience but also in interdisciplinary applications such as neuromarketing. These innovations offer deeper insights into brain function and cognitive processes in consumer behavior ( Guixeres et al., 2017; Garczarek-Bąk, 2019; Russo, Clement, Jin, Liu, & Zito, 2022). Despite the availability of various neuroimaging methods, PET and fMRI remain the most prominent tools specifically utilized in neuromarketing research due to their ability to directly measure brain activity associated with emotional and decision-making processes.

Neuromarketing seeks to uncover the subconscious neural mechanisms influencing consumer behavior. Unlike traditional marketing approaches that rely on self-reported data, neuromarketing offers deeper insights into decision-making by analyzing brain activity and non-conscious responses. As Nemorin et al. emphasize, the distinctiveness of neuromarketing lies in its ability to circumvent the conscious cognitive filters of consumers, instead accessing brain activity directly to derive more authentic insights ( Goncalves, Hu, Aliagas & Cerdá, 2024; Romanowski, 2019). Over the past five years, the majority of neuromarketing research has concentrated on two primary categories of stimuli: product-related cues (with or without price information) and promotional materials. In these studies, the term “product” encompasses both physical items such as tasting a beverage and conceptual depictions, like three-dimensional visual images. Price matters are frequently introduced as a key experimental variable, either independently or in conjunction with product or promotional content ( Nemorin, 2016; Taqwa et al., 2015). This pricing information significantly affects consumer decision-making by activating specific cognitive and emotional responses. Empirical findings based on event-related potentials (ERPs) suggest that price promotions can enhance the perceived value of certain high-priced yet affordable luxury products, thereby increasing consumers’ intentions to purchase ( Nemorin, 2016). Additionally, neuromarketing research has demonstrated that manipulations in pricing can alter neural correlates of experienced pleasure. Research found that increasing the price of wine led participants to report greater flavour enjoyment, a response corroborated by heightened activity in brain regions linked to sensory pleasure and reward processing ( Spence, 2024).

Neuromarketing techniques, especially those utilizing advanced neuroimaging tools, are transforming how brands decode and influence consumer behaviour in today’s attention-driven economy. Beyond merely supplementing traditional methods, these approaches reveal real-time neural responses associated with emotional engagement, brand perception, and decision heuristics offering marketers evidence-based pathways to optimize campaign effectiveness ( Plassmann et al., 2019). The various application of advanced techniques allows marketers to pinpoint the neural correlates of attention, memory encoding, reward processing, and emotional engagement ( Petrovic, Petersson, & Ingvar, 2021; Venkatraman et al., 2012; Hasnaoui, Benabdallah, & Djebbari, 2023). These approaches are not only scientifically rigorous but also practically valuable, enabling firms to refine product designs, price strategies, and promotional content in ways that resonate deeply with target audiences ( Ariely & Berns, 2010; Chen & Hsu, 2023). For instance, fMRI research has been used to anticipate the success of advertising campaigns and music singles before their public release, indicating the strong real-world applicability of neuromarketing ( Berns & Moore, 2012; Wivedi, Sharma, 2024). While concerns about ethical application and methodological rigor are valid, the field has made considerable strides in standardizing protocols and addressing biases. As such, leveraging diverse and validated neuromarketing techniques remains a promising and legitimate pathway to achieving strategic marketing goals provided that methods are carefully selected and ethically implemented ( Rawnaque, Rahman & Anwar, 2020; Morin, 2011). Studies reveal that consumer attitudes and behavioural responses are shaped by unconscious neural developments, often inaccessible through self-reports ( Hasnaoui, Benabdallah, & Djebbari, 2023). The advance configurated techniques like fMRI, EEG and other which enhance prediction accuracy for campaign effectiveness. These intellectual insights enable marketers to tailor strategies more precisely, bridging gaps left by traditional methods and improving engagement consequences.

## Conventional to sophisticated insights of neuromarketing

The conventional approach to market research and data collection, aimed at fulfilling business requirements, proves inadequate in delivering the desired outcomes. Consequently, the adoption of novel techniques and methodologies for acquiring reliable evidence has become authoritative. To offset the substantial costs of advertising campaigns often undertaken by companies to promote new products or increase sales of existing one’s financial returns or measurable market impact are typically required to justify the investment ( Şik & Soba, 2021; Yang, Zhang, Qian, & Wang, 2022). It is widely acknowledged that the success of advertisements cannot be fully captured through verbal self-reports alone. Attempting to interpret emotional responses purely through cognitive or linguistic frameworks often leads to incomplete or misleading conclusions ( Plassmann et al., 2015). Emotions are not solely grounded in language they are complex affective states that may elude conscious articulation. As such, accurately assessing emotional engagement requires moving beyond verbal expression to incorporate neurocognitive and physiological measures, which offer deeper insights into how consumers truly respond to advertising stimuli. Furthermore, such evaluations are subject to criticism for failing to capture the relationship between the influence of advertisements and subsequent consumer behavior ( Andreassi, 2007; Ariely & Berns, 2010; Romanowski, 2019). Consumer preferences, decisions, and choices are frequently influenced through Neuromarketing techniques. Despite businesses being hesitant to disclose the utilization of Neuromarketing tactics to enhance their marketing efforts, there have been a limited number of extensively documented research studies in this field. Various studies have indicated that the presence of attractive celebrities in advertisements stimulates specific brain regions, leading to the recognition of the product and the development of inner trust ( Hubert & Kenning, 2008; Khushaba, Wise, Kodagoda, Louviere, Kahn, & Townsend, 2019). In addition, customers are inclined to associate specific advertising elements with certain situations who respond strongly and make purchases accordingly. These studies enable businesses to shape, modify, and select advertisements in a manner that effectively resonates with and remains in the memory of their clients. The Specific marketing allows businesses to identify the neurological factors that consumers are engaged with in advertising. This provides a deeper understanding of how language, visuals, sound effects, and music can be utilized to create more compelling advertisements for their target audience ( Gholami, 2024). Marketing managers have access to two sources of information that are crucial for making decisions: conventional research techniques and Neuromarketing techniques. The use of psychophysiological estimations allows participants to form preferences and make decisions through conscious thought, as opposed to relying on self-reported estimations of emotional states and unconscious cognitive processes that influence consumer responses in marketing studies. In other words, this approach goes beyond conscious aspects of human behavior and can identify influences on behavior even when consumers are not aware of them ( Zaltman, 2003; Ko, Kim, & Lee, 2021). This made significant contribution to consumer research, considering that approximately 95% of our cognitive and emotional processing occurs at unconscious levels.

Currently, state-of-the-art eye-tracking systems and electrooculography (EOG) a technique that records eye movements by measuring the electrical potential between the front and back of the eye enable the detection of subtle changes in visual attention and ocular behavior. These technologies offer high temporal resolution, often within just a few milliseconds, specifically in tracking eye movement dynamics ( Duchowski, 2007), allowing researchers to precisely map attention patterns in real time. This is particularly useful for studying the effects of momentarily stimulating marketing stimuli and monitoring transient shopping behavior without impeding the decision-making process ( Solnais, Andreu-Perez, Sanchez-Fernandez & Andreu-Abela, 2013). Electroencephalography (EEG) can be integrated with other advanced real-time determination techniques to provide additional insights into customer attention and interest ( Hasnaoui, Benabdallah, & Djebbari, 2023). It can also be utilized in combination with other high time resolution technologies to provide more comprehensive information about viewer attention and arousal during advertising. For example, by synchronizing data from EEG recordings, it is possible to identify the precise focus of the member’s attention at a given moment in time, as well as the specific elements of the scene that triggered activation of the left frontal hemisphere ( Nemorin, 2016). The overall benefits of these technologies contribute to the increasing popularity of Neuromarketing and consumer neuroscience for advertising testing. Neuromarketing utilizes state-of-the-art resources in brain scanning to understand the customer’s purchasing behavior. Schneider & Woolgar (2012) assert that Neuromarketing is the latest tool employed by marketing researchers to comprehend consumer behavior. In fact, understanding consumer behavior, particularly the decision-making processes involved in purchasing, emerges as one of the most frequently cited objectives in neuromarketing research ( Eser et al., 2011; Suhendra, Hermita, & Darmayantie, 2015; Yang, Zhang, Qian, & Wang, 2022). Lee et al. (2007) also states that Neuromarketing has become a popular technology for determining the likelihood and non-probability of purchasing decisions, which has also been identified as a means of shaping companies’ marketing strategies ( Lee et al., 2007; Eser et al., 2011, Nemorin, 2016). Additionally, it helps to eliminate elements that should not be present in the communication, such as elements that lead to consumer aversion to the products. It also assists in the selection of visual and auditory features, as well as the timing and choice of appropriate media. Neuromarketing also has the ability to identify consumers’ needs and, consequently, develop more meaningful and comprehensive consumer marketing strategies.

## Non-invasive phenomena for understanding consumer behaviours

Advances in neuroimaging and cognitive neuroscience have facilitated the use of non-invasive techniques such as EEG, fMRI, MEG, and eye-tracking, among other biometric tools to decode consumer behavior with remarkable precision. These tools reveal unconscious emotional and cognitive processes, complement traditional methods by providing deeper insights, and establish a scientific foundation for evidence-based marketing interventions ( Khushaba et al., 2019; Yang, Zhang, Qian, Wang, 2022; Hsu & Yoon, 2023). These tools reveal unconscious emotional and cognitive processes, complements deeper insights than traditional methods ( Khushaba, et al., 2019; Yang, Zhang, Qian, & Wang; 2022; Hsu & Yoon, 2023), and establishing a scientific foundation for evidence-based marketing interventions. Central to this shift is the application of non-invasive brain imaging techniques that offer a powerful means for locating neural activity in response to marketing stimuli, allowing researchers and practitioners to decode how consumers prioritize, evaluate, and select products in complex environments. Among the most utilized non-invasive techniques, EEG stands out for its temporal resolution, portability, and cost-efficiency ( Vecchiato et al., 2011), argue that EEG enables detailed observation of cognitive and emotional processing, particularly through measurements of event-related potentials (ERPs) and frontal asymmetry ( Vecchiato et al., 2011; Mileti, Guido, & Prete, 2016). For instance, increased activity in the left prefrontal cortex is continuously associated with approach behaviour and positive evaluation, making it a key biomarker for identifying consumer preferences. As point out by Khushaba et al. (2013), EEG is particularly effective in contexts where consumers’ conscious reasoning does not fully account for their final purchase decisions such as during exposure to emotionally charged advertisements or branding content.

Further expanding the scope of consumer behavior analysis, explore the theoretical and ethical boundaries of neuromarketing in comparison to “mind-reading.” They emphasize that while non-invasive methods cannot access private thoughts, they can reliably capture automatic reactions that correlate with preference and motivation ( Booth & Freeman, 2014). In particular, neurophysiological responses to product packaging, advertisement narrative arcs, or price visibility provide marketers with cues about what drives attention and valuation, thereby aiding in more precise campaign tailoring. Complementing the neurophysiological focus, Medina et al. (2020) employed neuroimaging tools to compare prosocial and non-prosocial consumer responses during pricing decisions. This study found that the insular and prefrontal regions show differentiated activation depending on ethical and prosocial dispositions. The implication here is that consumer priorities are not merely rational or economic but also neurologically modulated by identity-related values and social cognition, and these can be decoded using non-invasive methods like fMRI and EEG in combination.

An emerging and increasingly significant development within neuromarketing is the use of real-time neural data to drive highly personalized marketing strategies, which Mileti et al. (2016) describe as an advanced form of neuroadaptive marketing a specialized subset that dynamically tailors advertising content based on immediate neural responses. The neuroscience technique leverages consumer micro-signals, such as moment-to-moment emotional fluctuations detected by facial coding or eye-tracking, in conjunction with EEG signals to anticipate behavioral outcomes ( Mileti et al., 2016; Yang, Zhang, Qian, & Wang, 2022). This hyper-individualized approach has profound implications for e-commerce and dynamic pricing models, allowing for the adaptation of product offers in milliseconds based on neural correlates of interest or aversion. The broader economic relevance of integrating neuromarketing into innovation management becomes particularly apparent when addressing uncertain consumer preferences in new product development ( Romanowski, 2019). Further, non-invasive tools allow for early testing of prototypes, packaging, or pricing strategies, thereby reducing market failure risk. The strategic integration of neuromarketing within product innovation pipelines represents a methodological leap, embedding consumer intuition directly into corporate decision-making. The extensive non-invasive neuromarketing tools provide a scientifically grounded and ethically feasible method for uncovering the latent processes that shape consumer priorities. They circumvent the biases inherent in self-reported data, provide an objective basis for segmentation, targeting, and positioning. However, minor challenges remain in standardizing protocols, ensuring data interpretability, and maintaining consumer consent and transparency. Nonetheless, the field’s trajectory supported by a growing body of evidence signals a development toward becoming an indispensable facet of modern consumer insight generation.

## Neuroscience approaches for mapping consumer behaviour and choices

Traditional marketing tools often fail to capture the subconscious cognitive and emotional mechanisms that shape consumer behavior ( Pradeep et al., 2022; Genevsky & Knutson, 2020). In contrast, neuromarketing leverages advanced non-invasive techniques such as EEG, fMRI, MEG etc. to observe neural responses in real-time, without requiring conscious effort from participants. These approaches allow researchers to pinpoint brain regions activated during brand exposure, decision-making, and emotional engagement, thus offering granular insights that surpass those obtained from self-reported data. Recent studies demonstrate that these neuroscientific methods significantly enhance the predictive validity of consumer responses, enabling marketers to design more effective strategies based on neurobiological evidence ( Morin et al., 2023).

Neuromarketing is a highly influential marketing strategy in the contemporary world. The possibility and the impact of these techniques investigated using various methods and approaches ( Stanton et al., 2017). The effective new methods are rooted in neuroscience, and neuroimaging techniques play a crucial role in testing hypotheses, enhancing existing knowledge, and examining the effects of marketing stimuli on consumers’ brains. Neuro marketing strategies can be categorized into three broad categories: recording metabolic activities in the brain, recording electrical activity in the brain, and which exploring brain activity without recording. Each of these categories differs in nature and serves as significant parameters for exploring the intentions, thoughts, hesitations, and other influential factors within the marketing industry.

## Electroencephalography

Electroencephalography (EEG) is a highly effective and cost-efficient technique for investigating the dynamics between brain activity and behavior ( Bell & Cuevas, 2012). Notably, the examination of various spectral bands, including Delta (0– Hz), Theta (3–7 Hz), Alpha (8–12 Hz), Beta (13–30 Hz), and Gamma (30– 0 Hz), has been employed to analyze consumers’ cognitive and affective processes in response to marketing stimuli ( Mostafa, 2012). The versatility of this method allows researchers to explore an extensive range of fields, enabling a comprehensive understanding of the temporal sequencing of neural events ( Light et al., 2010). The recording of EEG signals is conducted by placing multiple electrodes on the participant’s scalp in order to enhance the conduction of impulses to the electrodes. The electric current generated by the typical human brain is on the order of a few microvolts. These voltage fluctuations are a result of ionic currents flowing between the brain and nerve cells. Nerve cells communicate with each other through electrical impulses ( Mileti, Guido, & Prete, 2016; Yang, Zhang, Qian, & Wang, 2022; Hsu & Yoon, 2023). The electrical potential detected by an individual neuron is extremely weak and therefore not detectable. Each electrode reflects the combined activity of thousands or millions of neurons with the same spatial orientation, thus the EEG measures the overall synchronous activity of a large number of neurons present in the brain. It is highly valuable in evaluating emotional stability and monitoring emotional conditions ( Hsu & Yoon, 2023; Kline, 2000). This device is a portable tool that aids in assimilating and synchronizing stimuli. The only limitation of this method is that it can only record apparent electrical signals and cannot differentiate deep brain structures or exchange information. Consequently, the EEG provides greater temporal resolution and less spatial resolution in the field of neuroscience. The electroencephalogram technology, frequently utilized in neuromarketing, offers insightful data on brain activity and is both portable and reasonably priced.

Advanced wireless electroencephalogram device represents a significant breakthrough in neuromarketing to enabling real-time, mobile, and ecologically valid monitoring of brain activity in naturalistic environments. These devices offer high temporal resolution and improved comfort, allowing researchers to track consumers’ neural responses to advertisements, retail layouts, and digital content outside laboratory constraints ( Ma et al., 2022; Luis-Alberto, & Sanchez-Fernandez, 2022). The effectiveness in capturing emotional engagement, cognitive load, and attention metrics during real-world consumer experiences. As wearable EEG technology becomes more sophisticated, it opens new possibilities for continuous consumer insight, adaptive advertising, and personalized marketing strategies based on neurophysiological feedback.

## Positron emission tomography

Positron Emission Tomography (PET) is a highly prominent technique in the field of Neuromarketing ( Song et al., 2025). It is a three-dimensional diagnostic imaging method that utilizes Positron-emitting radioisotopes to assess messenger cell metabolism. Through injecting radioactive elements (positrons) into participants, these elements mix with the blood and pass through the brain. However, it is worth noting that PET is an invasive method and is less commonly used related to fMRI, PET has the capability to measure sensory perception and the emotional valence in response to marketing stimuli. The techniques achieve this by employing radio labelled molecules to visualize the molecular interactions of biological processes. The principle behind PET imaging involves the detection of two high-energy photons emitted simultaneously from a positron-emitting radioisotope. According to Vaquero and Kinahan (2015), PET offers superior translation capabilities compared to other methods due to its combination of sensitivity and quantitative accuracy ( Vaquero & Kinahan, 2015). Similarly, Jones and Townsend (2017) suggest that PET is the most specific and sensitive means of imaging molecular interactions and pathways in humans ( Jones & Townsend, 2017). These techniques are helps to observe which brain areas activate when consumers view brands or advertisements, helping marketers comprehend emotional engagement and preference by tracking activity in zones like the prefrontal cortex and reward centers.

## Functional magnetic resonance imaging

Functional Magnetic Resonance Imaging (fMRI) is widely regarded as the primary method for investigating. This technique allows for the analysis of intricate and minute brain structures, in contrast, scanning is costly compared to other methods. there is a delay of 6-10 seconds in recording neural activity, which poses a limitation when measuring marketing stimuli ( Ariely & Berns, 2010). The high cost of fMRI scanning is primarily due to the small sample size used for analysis. There are two main techniques used in functional MRI: the Blood Oxygen Level Dependent (BOLD) technique and the dynamic exogenous technique ( Hare et al., 1998). The BOLD technique is preferred as it does not require intravenous contrast. Chow et al. (2017) highlight that the majority of fMRI studies utilize BOLD contrast imaging, which involves mapping active regions of the brain based on changes in blood oxygen. Blood flow in the brain is locally regulated in response to oxygen and carbon dioxide levels in cortical tissue. This method has garnered significant attention in the field of Neuromarketing. While fMRI provides excellent spatial resolution, its temporal resolution is relatively poor. In Neuromarketing, fMRI is employed to measure various factors such as memory encoding, emotional valence, sensory perception, brand loyalty and trust, brand preference, and brand recall.

Recent advancements have leveraged fMRI to study neural valuation systems particularly activity in the ventromedial prefrontal cortex (vmPFC) and nucleus accumbens as reliable predictors of purchasing intention and product desirability ( Enax et al., 2015; Plassmann et al., 2021). Furthermore, emerging fMRI-based research explores the brain’s response to sustainability messaging and ethical branding, revealing heightened activation in areas linked to moral reasoning and social cognition, such as the temporoparietal junction and dorsomedial prefrontal cortex ( Karmarkar, Bollinger, 2022). This study provides a new dimension to understanding consumer behaviour, where decision-making is influenced not only by price and utility, but also by values, emotional resonance, and ethical alignment. As fMRI continues to evolve with improved models and machine learning integration, it offers deeper insights into the subconscious processes driving consumer preferences in increasingly complex marketplaces.

## Transcranial Magnetic Stimulation

Transcranial Magnetic Stimulation (TMS) is a non-invasive neuroscience technique used to stimulate targeted areas of the cerebral cortex which enables researchers to establish causal links between specific brain regions and behavioural responses to inducing temporary changes in neural activity. The devise placed to the scalp, TMS generates a magnetic field that stimulates underlying brain tissue, unsettling or enhancing cortical processing in localized areas ( Sliwinska, Vitilo & Devlin, 2014). This permits for precise manipulation of cognitive functions and provide insights into the timing and role of neural processes in real-time decision-making. In addition, consumer research, TMS provides a powerful technique to explore how the brain responds to marketing stimuli. Through selectively activating or inhibiting regions associated with emotion, attention, or decision-making, researchers can assess how these changes effect purchasing attitudes and behavior. Namely, recent studies using TMS on the dorsolateral prefrontal cortex (DLPFC) disclose that modulation of this region can significantly alter consumer assessment and risk preferences in purchase decisions ( Gupta et al., 2021). Moreover, this technique has been effectively used to study the influence of affective priming on product judgments, indicating that temporary disruption of emotion-processing circuits can modulate brand attitudes and consumer trust ( Zhang & Li, 2022). Research findings support the growing role of TMS in neuromarketing, where it helps decode, the neural mechanisms underlying consumer attention, preference formation, and decision-making, enabling marketers to develop empirically informed engagement strategies.

Indeed, TMS can directly influence consumption-related behaviours, such as delaying gratification or shifting preference toward serviceable versus hedonic products ( Ramsoy et al., 2021). Furthermore, emerging TMS applications in neuromarketing explore the modulation of empathy and social cognition through ethical consumption decisions, revealing that stimulating the right temporoparietal junction can alter perceived brand authenticity and trust ( Cian et al., 2022). Through effectively use these techniques is not merely a validation tool for neural correlates but a mechanism for probing and reshaping the cognitive architecture of consumer thought itself. This opens promising avenues for designing interventions in areas such as compulsive buying, financial risk behavior, and ethical advertising.

## Steady State Topography

Steady-State Topography (SST), introduced by Richard Silberstein in 1990, is a cutting-edge neurophysiological method for mapping neural activity in response to external stimuli. Initially developed for cognitive neuroscience, this device supports significant applications in neuromarketing and consumer neuroscience, especially in evaluating consumer perception, emotive engagement, and brand communication ( Maran, Moonisha, Patel, Mari Muthu, & Anbazhagan, 2021). In SST experiments, participants are exposed to audiovisual stimuli while a dim sinusoidal flicker is presented in their peripheral vision, causing a steady-state visually evoked potential (SSVEP). This neural response is measured across cortical areas to assess variations in cognitive and emotional processing. The specific concern of SST particularly, valuable in neuromarketing is its sensitivity to changes in latency the delay between stimulus and neural response which reflects long-term memory encoding, sustained attention, and emotional intensity ( Silberstein & Nield, 2021). Through analysing latency shifts and cortical activation patterns over time, SST enables a more nuanced understanding of how consumers perceive advertising messages, process branding cues, and form durable attitudes toward products. Steady-State Topography based new research demonstrated its ability to predict advertising effectiveness and product recall with high precision, making it an advanced tool for capturing subconscious consumer behavior in real-world media environments.

## Magnetoencephalography

Magnetoencephalography (MEG) is a technique that measures brain activity by detecting potential magnetic fields on the scalp using sensitive detectors housed in a helmet placed on the head. Unlike other methods, MEG is not influenced by tissue type, such as blood, brain matter, or bone, and can provide spatial and temporal resolution to determine the depth of a point in the brain. The MEGs are capable of monitoring population neuronal activity in the brain, but the costs associated with their use are significant due to the requirement for a magnetic field-free environment. This device records electrical activity in the brain, offering high temporal resolution to trace the consumers’ responses to marketing stimuli in real time.

The Magnetoencephalography offer unique advantage in neuromarketing method through providing millisecond-level temporal resolution and superior spatial localization of brain activity compared to Electroencephalography, while remaining non-invasive unlike functional magnetic Resonance. This method is very magnificent to captures magnetic fields generated by neuronal activity which allowing precise tracking of consumer responses to marketing stimuli such as advertisements and product designs ( Lee et al., 2021; Morinaga et al., 2022; Dikker & Pineda, 2023). Undouble this unique method effectively maps cognitive processes like brand recall, emotional arousal, and decision-making pathways. This real-time, high-fidelity insight supports profound consumer profiling and enhances predictive modelling in marketing strategies.

## Conclusion

Recent innovations in neuroscience and marketing have significantly transformed consumer behaviour research, enabling a deeper understanding of the subconscious drivers of decision-making. By integrating biologically grounded and temporally precise insights, these methods address the limitations of traditional self-report measures. Non-invasive techniques such as EEG, PET, SST, and fMRI have expanded methodological boundaries by allowing real-time tracking of attention, memory encoding, and emotional responses in ecologically valid environments. However, these advancements come with notable challenges, including variability across devices, inconsistent preprocessing protocols, and the inferential complexity of linking neural signals to psychological constructs like preference or intent. The growing use of machine learning models, especially Support Vector Machines (SVMs) and deep learning approaches, has enhanced analytical power but also raised concerns regarding model transparency and generalizability. As commercial applications of neuromarketing expand, ethical considerations around informed consent, data privacy, and cognitive manipulation become increasingly urgent. To establish neuromarketing as a scalable and ethically responsible discipline, future research must focus on methodological standardization, improved interpretability, and critical reflection on the broader implications of decoding consumer cognition

## Data Availability

All data are publicly available and cited in the manuscript. No new data were generated, and no DOI applies.
