# The Neuroscience of Marketing: Noninvasive Neuromarketing Methods for Understanding Consumer Choice and Priorities

**DOI:** 10.12688/f1000research.168220.4

**Published:** 2026-08-29

**Authors:** Pradeep K, Sathya Prasad P.V, Rajalakshmi S A, Chriso Ricky Gill J, Jisha V. G

**Affiliations:** 1 Department of Media Studies, Christujayanthi University (DU), Bengaluru, India; 2 Department of Social Work, Amrita School of Social and Behavioural Sciences, Amrita Vishwa Vidyapeetham, Coimbatore, Tamilnadu, 641112, India; 3 Department of English, Vel Tech High Tech Dr. Rangarajan Dr. Sakunthala Engineering College, Avadi, India; 4 Department of English, Noorul Islam Centre for Higher Education, Kumaracoil, Thuckalay, Tamilnadu, India

**Keywords:** Neuroscience of Marketing, Neuromarketing, Consumer behaviour, Marketing, non-invasive techniques

## Abstract

Neuromarketing and consumer neuroscience aim to enhance self-report and behavioral techniques by assessing neurophysiological and psychophysiological reactions that are frequently challenging to quantify. This study consolidates non-invasive tools frequently employed to examine consumer reactions to marketing stimuli, emphasizing the metrics each method assesses, the marketing inquiries it may reliably address, and the potential dangers of inference involved. We systematically compile evidence pertaining to constructs fundamental to consumer choice, including attention, affective arousal, memory encoding, and valuation, while comparing methodologies such as EEG, fMRI, TMS, Steady State Topography and MEG. The article proposes a method selection matrix that associates common research and applied inquiries (e.g., advertising attention dynamics, packaging assessment, pricing, and value indicators) with suitable measurement strategies, highlighting trade-offs concerning temporal/spatial resolution, ecological validity, cost, and interpretability. Finally, examine methodological challenges and ethical obligations. The scoping review concludes with research gaps where multimodal and more ecologically valid designs are most likely to advance the field.

## Introduction

Marketing decisions today depend on consumers’ quick, automatic responses to complex stimuli, such as short-form video ads, interactive shopping interfaces, and optimized product packaging ( Lin et al., 2018; Morin, 2011). Traditional self-reported methods, including surveys, interviews, and focus groups, are crucial for comprehending expressed beliefs and preferences. However, these methods are constrained by recall bias, demand characteristics, and consumers' limited introspective access to the cognitive and emotional processes that influence attention, emotion, and decision-making ( Pradeep et al., 2022). As a result, critical aspects of consumer decision-making often remain inaccessible through introspective methods alone.

Consumer neuroscience and its applied extension, neuromarketing, have emerged over the past two decades as a complementary framework aimed at addressing these limitations by integrating behavioural paradigms with noninvasive neurophysiological and psychophysiological measurements ( Lin et al., 2018; Clithero, Carter & Huettel, 2024). Techniques such as electroencephalography (EEG), functional magnetic resonance imaging (fMRI), functional near-infrared spectroscopy (fNIRS), eye tracking, and peripheral autonomic measures enable the examination of attention allocation, memory encoding, affective arousal, and valuation as they unfold in real-time ( Rawnaque et al., 2020). These methods are increasingly employed in marketing research because they can uncover consumer responses that frequently occur beyond conscious awareness. They offer time-sensitive and objective indicators of the underlying cognitive processes ( Mohd Isa & Anuar, 2024). Notably, neuromarketing is not intended to replace traditional methods; instead, it serves as a complementary approach that enhances the explanatory power through triangulation by integrating subjective reports, observed behaviour, and physiological responses ( Morin, 2011).

Despite its growth and increasing visibility, neuromarketing research still faces conceptual, methodological, and ethical challenges that hinder its overall impact. Previous reviews have often followed a technique-by-technique approach, providing descriptive overviews of individual tools without offering sufficient synthesis across methods or marketing-relevant constructs ( Clithero et al., 2024). Persistent concerns regarding reverse inference, ecological validity, reproducibility, and ethical governance further complicate interpretation and application ( Fisher et al., 2010; Gonçalves et al., 2024). Addressing these gaps, the present review focuses explicitly on noninvasive neuromarketing methods and contributes by (i) proposing a clear typology of commonly used neurophysiological and psychophysiological measures; (ii) offering an integrative comparison linking key marketing questions, such as attention capture, emotional engagement, memory formation, and valuation, to appropriate tools and their limitations; and (iii) synthesising evidence to distinguish what is currently robust from what remains uncertain. By adopting this construct-centred and method-comparative perspective, this review aims to advance both scientific rigor and responsible application in contemporary marketing research ( Lin et al., 2018; Rawnaque et al., 2020). This review explores neuromarketing as a developing interdisciplinary method aimed at elucidating consumer choices and priority development. Further, demonstrates how the integration of neurophysiological evidence with marketing theory and ethical standards can improve the comprehension of automatic and context-dependent decision-making processes in modern consumer environments.

## Integrative review trajectories in consumer decision studies

This article pursued a narrative scoping review methodology to synthesise evidence on non-invasive neuromarketing methods and their applications in understanding consumer decision-making processes. The review aims to map the evolution of neuromarketing techniques, examine their associations with key consumer neuroscience constructs including attention, emotion, memory, and valuation, and identify methodological opportunities and limitations reported in the literature. Relevant publications were identified through systematic searches of major academic databases such as Scopus, Web of Science, PubMed, and Google Scholar. Literature published from 2000 to 2026 was included to capture both the emergence and recent advancements in neuromarketing research. Search terms comprised combinations of “neuromarketing,” “consumer neuroscience,” “EEG,” “fMRI,” “MEG,” “TMS,” “Steady-State Topography,” “eye tracking,” “psychophysiology,” “consumer decision-making,” “ttention,” “emotion,” “memory,” and “valuation.” The research were included if they investigated neuroscientific, physiological, or behavioural measurement approaches within marketing-related contexts, such as advertising evaluation, branding, product assessment, pricing decisions, and consumer choice. Studies focusing solely on clinical neuroscience or unrelated cognitive applications were excluded. The selected literature was analysed comparatively to develop an integrated framework that links measurement approaches with consumer-related constructs, practical marketing applications, and associated interpretive limitations.

## Understanding the consumer behaviour through neuroimaging paradigm

Currently, various sophisticated technologies are employed to map neural responses related to consumer behavior and other cognitive functions. Among these, two primary neuroimaging techniques functional magnetic resonance imaging (fMRI) and positron emission tomography (PET) are commonly used in neuroscience to visualize brain activity by assessing changes in blood flow. These methods are grounded in the neurophysiological principle that mental activity increases the demand for oxygen and glucose in activated brain regions a demand that is met through increased cerebral blood flow ( Bault & Rusconi, 2020; Kim, H. E. et al., 2022; Dwivedi & Sharma, 2024). fMRI detects brain activity by measuring changes in blood oxygen levels. When neurons become active, blood flow increases, delivering more oxygenated blood to the region. This influx reduces deoxygenated hemoglobin concentration, enhancing the signal and allowing high spatial resolution mapping of neural activity. The Positron emission tomography, on the other hand, involves the injection of radioactive tracers that emit positrons. These tracers such as oxygen-15 and fluorodeoxyglucose (18F-FDG), accumulate in regions of the brain exhibiting increased neural activity, which corresponds to elevated blood flow ( Rawnaque, Rahman, & Anwar, 2020; Alsharif & Mohd Isa, 2024). The resulting radiotracer emissions are detected by PET scanners to produce high-resolution images that reflect patterns of regional glucose metabolism an essential marker of brain function. Alterations in glucose metabolism often indicate underlying neurological or cognitive conditions. Rong, Lulong & Zhihua (2024) observed that medical imaging technologies are advancing rapidly, with molecular imaging emerging as a particularly promising field. The novel innovation in this area have sparked interest not only in clinical neuroscience but also in interdisciplinary applications such as neuromarketing. These innovations offer deeper insights into brain function and cognitive processes in consumer behavior ( Guixeres et al., 2017; Garczarek-Bąk, 2019; Russo, Clement, Jin, Liu, & Zito, 2022). Despite the availability of various neuroimaging methods, PET and fMRI remain the most prominent tools specifically utilized in neuromarketing research due to their ability to directly measure brain activity associated with emotional and decision-making processes.

Neuromarketing seeks to uncover the subconscious neural mechanisms influencing consumer behavior traditional marketing relies on consumers’ self-reported data, while neuromarketing uncovers deep insights into consumer decision-making by parsing brain activity and non-conscious responses; the core differences between these two approaches form the starting point of research in this field. Scholars including Nemorin et al. propose that neuromarketing can bypass consumers’ conscious cognitive filters to obtain more authentic decision-making information ( Romanowski, 2019; Goncalves et al., 2024). Over the past five years, neuromarketing research has focused on two categories of stimuli: product cues and promotional materials, each presented either with or without price information. In these studies, the term “product” covers both physical goods (such as beverage tasting trials) and conceptual presentations (such as 3D visual images). As a core experimental variable, price is set in two ways: as an independent standalone variable, or integrated with product or promotional content. Relevant foundational studies include the 2016 work by Nemorin and the 2015 study by Taqwa et al. Empirical research based on event-related potentials (ERPs) ( Nemorin, 2016; Gorin et al., 2025) shows that price promotions can increase the perceived value of high-priced yet affordable luxury goods, thereby boosting consumers’ purchase willingness.

The brain centric techniques, especially those utilizing advanced neuroimaging tools, are transforming how brands decode and influence consumer behaviour in today’s attention-driven economy. Beyond merely supplementing traditional methods, these approaches reveal real-time neural responses associated with emotional engagement, brand perception, and decision heuristics offering marketers evidence-based pathways to optimize campaign effectiveness ( Plassmann et al., 2019). The various application of advanced techniques allows marketers to pinpoint the neural correlates of attention, memory encoding, reward processing, and emotional engagement ( Petrovic, Petersson, & Ingvar, 2021; Venkatraman et al., 2012; Hasnaoui, Benabdallah, & Djebbari, 2023). These approaches are not only scientifically rigorous but also practically valuable, enabling firms to refine product designs, price strategies, and promotional content in ways that resonate deeply with target audiences ( Chen & Hsu, 2023). For instance, fMRI research has been used to anticipate the success of advertising campaigns and music singles before their public release, indicating the strong real world applicability of neuromarketing ( Berns & Moore, 2012; Dwivedi & Sharma, 2024). While concerns about ethical application and methodological rigor are valid, the field has made considerable strides in standardizing protocols and addressing biases. As such, leveraging diverse and validated neuromarketing techniques remains a promising and legitimate pathway to achieving strategic marketing goals provided that methods are carefully selected and ethically implemented ( Rawnaque, Rahman & Anwar, 2020; Morin, 2011). Studies reveal that consumer attitudes and behavioural responses are shaped by unconscious neural developments, often inaccessible through self-reports ( Hasnaoui, Benabdallah, & Djebbari, 2023). The advance configurated techniques like fMRI, EEG and other which enhance prediction accuracy for campaign effectiveness. These intellectual insights enable marketers to tailor strategies more precisely, bridging gaps left by traditional methods and improving engagement consequences.

## Conventional to sophisticated insights of neuromarketing

The conventional approach to market research and data collection, aimed at fulfilling business requirements, proves inadequate in delivering the desired outcomes. Consequently, the adoption of novel techniques and methodologies for acquiring reliable evidence has become authoritative. To offset the substantial costs of advertising campaigns often undertaken by companies to promote new products or increase sales of existing one’s financial returns or measurable market impact are typically required to justify the investment ( Şik & Soba, 2021; Yang, Zhang, Qian, & Wang, 2022). It is widely acknowledged that the success of advertisements cannot be fully captured through verbal self-reports alone. Attempting to interpret emotional responses purely through cognitive or linguistic frameworks often leads to incomplete or misleading conclusions ( Plassmann et al., 2015). Emotions are not solely grounded in language they are complex affective states that may elude conscious articulation. As such, accurately assessing emotional engagement requires moving beyond verbal expression to incorporate neurocognitive and physiological measures, which offer deeper insights into how consumers truly respond to advertising stimuli. Furthermore, such evaluations are subject to criticism for failing to capture the relationship between the influence of advertisements and subsequent consumer behavior ( Romanowski, 2019). Consumer preferences, decisions, and choices are frequently influenced through Neuromarketing techniques. Despite businesses being hesitant to disclose the utilization of Neuromarketing tactics to enhance their marketing efforts, there have been a limited number of extensively documented research studies in this field. Previous studies by Hubert and Kenning (2008) and Khushaba et al. (2019) point out that appealing celebrity advertisements can activate specific brain regions in consumers, driving consumers to complete product recognition and build inherent trust in products. Gholami (2024) further found that consumers also proactively link specific advertising elements to corresponding scenarios, generate strong responses, and ultimately complete purchases. Such studies can help enterprises screen and adjust advertisements that fit consumer needs and can be retained in audiences’ memories, accurately identify factors that trigger neural responses, and reasonably leverage elements including language, visuals, sound effects, and music to create targeted advertisements. The core decisions of marketing managers rely on two types of information sources: traditional research technologies and neuromarketing technologies. The use of psychophysiological estimations allows participants to form preferences and make decisions through conscious thought, as opposed to relying on self-reported estimations of emotional states and unconscious cognitive processes that influence consumer responses in marketing studies. In other words, this approach goes beyond conscious aspects of human behavior and can identify influences on behavior even when consumers are not aware of them ( Zaltman, 2003; Ko, Kim, & Lee, 2021). This made significant contribution to consumer research, considering that approximately 95% of our cognitive and emotional processing occurs at unconscious levels.

Currently, state-of-the-art eye-tracking systems and electrooculography (EOG) a technique that records eye movements by measuring the electrical potential between the front and back of the eye enable the detection of subtle changes in visual attention and ocular behavior. These technologies offer high temporal resolution, often within just a few milliseconds, specifically in tracking eye movement dynamics allowing researchers to precisely map attention patterns in real time ( Duchowski, 2007; Khairunnisa & Sari, 2023). This is particularly useful for studying the effects of momentarily stimulating marketing stimuli and monitoring transient shopping behavior without impeding the decision-making process ( Solnais, Andreu-Perez, Sanchez-Fernandez & Andreu-Abela, 2013). Electroencephalography (EEG) can be integrated with other advanced real-time determination techniques to provide additional insights into customer attention and interest ( Hasnaoui, Benabdallah, & Djebbari, 2023). It can also be utilized in combination with other high time resolution technologies to provide more comprehensive information about viewer attention and arousal during advertising. For example, by synchronizing data from EEG recordings, it is possible to identify the precise focus of the member’s attention at a given moment in time, as well as the specific elements of the scene that triggered activation of the left frontal hemisphere ( Nemorin, 2016). The overall benefits of these technologies contribute to the increasing popularity of Neuromarketing and consumer neuroscience for advertising testing. Neuromarketing utilizes state-of-the-art resources in brain scanning to understand the customer’s purchasing behavior. Schneider & Woolgar (2012) assert that Neuromarketing is the latest tool employed by marketing researchers to comprehend consumer behavior. In fact, understanding consumer behavior, particularly the decision-making processes involved in purchasing, emerges as one of the most frequently cited objectives in neuromarketing research ( Eser et al., 2011; Suhendra, Hermita, & Darmayantie, 2015; Yang, Zhang, Qian, & Wang, 2022). Lee et al. (2007) also states that Neuromarketing has become a popular technology for determining the likelihood and non-probability of purchasing decisions, which has also been identified as a means of shaping companies’ marketing strategies ( Lee et al., 2007; Eser et al., 2011, Nemorin, 2016). Additionally, it helps to eliminate elements that should not be present in the communication, such as elements that lead to consumer aversion to the products. It also assists in the selection of visual and auditory features, as well as the timing and choice of appropriate media. Neuromarketing also has the ability to identify consumers’ needs and, consequently, develop more meaningful and comprehensive consumer marketing strategies.

## Non-invasive phenomena for understanding consumer behaviours

Recent advancements in neuroimaging and cognitive neuroscience have enabled the application of non-invasive techniques, including EEG, fMRI, MEG, and eye-tracking, alongside other biometric tools, to decode consumer behavior with notable precision. These methodologies uncover unconscious emotional and cognitive processes, thereby complementing traditional approaches by offering deeper insights and establishing a scientific basis for evidence-based marketing interventions ( Khushaba et al., 2019; Yang, Zhang, Qian, & Wang, 2022; Hsu & Yoon, 2023). Central to this paradigm shift is the use of non-invasive brain imaging techniques, which provide a robust means of identifying neural activity in response to marketing stimuli. This capability allows researchers and practitioners to decode consumer prioritization, evaluation, and selection of products within complex environments. Among these techniques, EEG is particularly prominent due to its temporal resolution, portability, and cost-effectiveness ( Raiesdana & Mousakhani, 2022; Vecchiato et al., 2011). EEG facilitates detailed observation of cognitive and emotional processing through metrics such as event-related potentials (ERPs) and frontal asymmetry ( Vecchiato et al., 2011; Mileti, Guido, & Prete, 2016). Notably, increased activity in the left prefrontal cortex is associated with approach behavior and positive evaluations, serving as a critical biomarker for consumer preferences. As highlighted by Khushaba et al. (2013), EEG proves particularly effective in scenarios where consumers' conscious reasoning does not fully explain their purchase decisions, such as during exposure to emotionally charged advertisements or branding content.

Further expanding the scope of consumer behavior analysis, explore the theoretical and ethical boundaries of neuromarketing in comparison to “mind-reading.” They emphasize that while non-invasive methods cannot access private thoughts, they can reliably capture automatic reactions that correlate with preference and motivation ( Booth & Freeman, 2014). In particular, neurophysiological responses to product packaging, advertisement narrative arcs, or price visibility provide marketers with cues about what drives attention and valuation, thereby aiding in more precise campaign tailoring. Complementing the neurophysiological focus, Medina et al. (2020) employed neuroimaging tools to compare prosocial and non-prosocial consumer responses during pricing decisions. This study found that the insular and prefrontal regions show differentiated activation depending on ethical and prosocial dispositions. The implication here is that consumer priorities are not merely rational or economic but also neurologically modulated by identity-related values and social cognition, and these can be decoded using non-invasive methods like fMRI and EEG in combination.

An emerging and increasingly significant development within neuromarketing is the use of real-time neural data to drive highly personalized marketing strategies, which Mileti et al. (2016) describe as an advanced form of neuroadaptive marketing a specialized subset that dynamically tailors advertising content based on immediate neural responses. The neuroscience technique leverages consumer micro-signals, such as moment-to-moment emotional fluctuations detected by facial coding or eye-tracking, in conjunction with EEG signals to anticipate behavioral outcomes ( Mileti et al., 2016; Yang, Zhang, Qian, & Wang, 2022). This hyper-individualized approach has profound implications for e-commerce and dynamic pricing models, allowing for the adaptation of product offers in milliseconds based on neural correlates of interest or aversion. The broader economic relevance of integrating neuromarketing into innovation management becomes particularly apparent when addressing uncertain consumer preferences in new product development ( Romanowski, 2019). Further, non-invasive tools allow for early testing of prototypes, packaging, or pricing strategies, thereby reducing market failure risk. The strategic integration of neuromarketing within product innovation pipelines represents a methodological leap, embedding consumer intuition directly into corporate decision-making. The extensive non-invasive neuromarketing tools provide a scientifically grounded and ethically feasible method for uncovering the latent processes that shape consumer priorities. They circumvent the biases inherent in self-reported data, provide an objective basis for segmentation, targeting, and positioning. However, minor challenges remain in standardizing protocols, ensuring data interpretability, and maintaining consumer consent and transparency. Nonetheless, the field’s trajectory supported by a growing body of evidence signals a development toward becoming an indispensable facet of modern consumer insight generation.

## Neuroscience approaches for mapping consumer behaviour and choices

Traditional marketing tools often fail to capture the subconscious cognitive and emotional mechanisms that shape consumer behavior ( Pradeep et al., 2022; Genevsky & Knutson, 2020). In contrast, neuromarketing leverages advanced non-invasive techniques such as EEG, fMRI, MEG etc. to observe neural responses in real-time, without requiring conscious effort from participants. These methodologies enable researchers to identify specific brain regions activated during brand exposure, decision-making, and emotional engagement, thereby providing detailed insights that exceed those derived from self-reported data. These techniques substantially improve the predictive validity of consumer responses, allowing marketers to develop more effective strategies grounded in neurobiological evidence ( Morin et al., 2023).

Neuromarketing is a highly influential contemporary marketing strategy. A 2017 study by Stanton et al. shows that the academic community has adopted different methods to explore the feasibility and impacts of this type of technology ( Stanton et al., 2017). Neuroimaging, the core supporting technology rooted in neuroscience that underpins neuromarketing, can verify hypotheses, enrich existing knowledge, and analyze the impacts of marketing stimuli on consumers’ brains. Neuromarketing can be divided into three categories: technologies that record brain metabolic activity, technologies that record electrical brain activity, and technologies that explore brain activity without recording. All three categories serve as important parameters for uncovering core marketing-influencing factors such as consumers’ intent and hesitation.

## Electroencephalography

Electroencephalography (EEG) is a highly effective and cost-efficient technique for investigating the dynamics between brain activity and behavior ( Bell & Cuevas, 2012). Notably, the examination of various spectral bands, including Delta (0– Hz), Theta (3–7 Hz), Alpha (8–12 Hz), Beta (13–30 Hz), and Gamma (30–0 Hz), has been employed to analyze consumers’ cognitive and affective processes in response to marketing stimuli ( Mostafa, 2012). The versatility of this method allows researchers to explore an extensive range of fields, enabling a comprehensive understanding of the temporal sequencing of neural events ( Light et al., 2010). The recording of EEG signals is conducted by placing multiple electrodes on the participant’s scalp in order to enhance the conduction of impulses to the electrodes. The electric current generated by the typical human brain is on the order of a few microvolts. These voltage fluctuations are a result of ionic currents flowing between the brain and nerve cells. Nerve cells communicate with each other through electrical impulses ( Mileti, Guido, & Prete, 2016; Yang, Zhang, Qian, & Wang, 2022; Hsu & Yoon, 2023). The electrical potential detected by an individual neuron is extremely weak and therefore not detectable. Each electrode reflects the combined activity of thousands or millions of neurons with the same spatial orientation, thus the EEG measures the overall synchronous activity of a large number of neurons present in the brain. It is highly valuable in evaluating emotional stability and monitoring emotional conditions ( Hsu & Yoon, 2023; Kline, 2000). This device is a portable tool that aids in assimilating and synchronizing stimuli. The only limitation of this method is that it can only record apparent electrical signals and cannot differentiate deep brain structures or exchange information. Consequently, the EEG provides greater temporal resolution and less spatial resolution in the field of neuroscience. The electroencephalogram technology, frequently utilized in neuromarketing, offers insightful data on brain activity and is both portable and reasonably priced.

Advanced wireless electroencephalogram device represents a significant breakthrough in neuromarketing to enabling real-time, mobile, and ecologically valid monitoring of brain activity in naturalistic environments. These devices offer high temporal resolution and improved comfort, allowing researchers to track consumers’ neural responses to advertisements, retail layouts, and digital content outside laboratory constraints ( Ma et al., 2022; Luis-Alberto, & Sanchez-Fernandez, 2022). The effectiveness in capturing emotional engagement, cognitive load, and attention metrics during real-world consumer experiences. As wearable EEG technology becomes more sophisticated, it opens new possibilities for continuous consumer insight, adaptive advertising, and personalized marketing strategies based on neurophysiological feedback.

## Functional magnetic resonance imaging

Functional Magnetic Resonance Imaging (fMRI) is widely regarded as one of the primary methods for investigating brain activity. This technique enables the analysis of intricate and minute brain structures; however, it is more expensive than many other methods. Moreover, the 6–10 second delay in recording neural activity represents a limitation when measuring responses to marketing stimuli ( Ariely & Berns, 2010). There are two main techniques used in functional MRI: the Blood Oxygen Level Dependent (BOLD) technique and the dynamic exogenous technique ( Hare et al., 1998). The BOLD technique is preferred as it does not require intravenous contrast. Chow et al. (2017) highlight that the majority of fMRI studies utilize BOLD contrast imaging, which involves mapping active regions of the brain based on changes in blood oxygen. Blood flow in the brain is locally regulated in response to oxygen and carbon dioxide levels in cortical tissue. This method has garnered significant attention in the field of Neuromarketing, while fMRI provides excellent spatial resolution, its temporal resolution is relatively poor. In Neuromarketing, fMRI is employed to measure various factors such as memory encoding, emotional valence, sensory perception, brand loyalty and trust, brand preference, and brand recall.

Recent advancements have employed fMRI to investigate neural valuation systems, with particular focus on activity in the ventromedial prefrontal cortex (vmPFC) and nucleus accumbens, which serve as reliable indicators of purchasing intention and product desirability ( Enax et al., 2015; Plassmann et al., 2021). Additionally, emerging fMRI-based research examines the brain's response to sustainability messaging and ethical branding, demonstrating increased activation in regions associated with moral reasoning and social cognition, such as the temporoparietal junction and dorsomedial prefrontal cortex ( Karmarkar, Bollinger, 2022). This study introduces a novel perspective on consumer behaviour, highlighting that decision-making is influenced not only by price and utility but also by values, emotional resonance, and ethical alignment. Despite, advance in fMRI technology with enhanced models and machine learning integration, it provides deeper insights into the subconscious processes that drive consumer preferences in increasingly complex marketplaces.

## Transcranial Magnetic Stimulation

Transcranial Magnetic Stimulation (TMS) is a non-invasive neuroscience technique used to stimulate targeted areas of the cerebral cortex which enables researchers to establish causal links between specific brain regions and behavioural responses to inducing temporary changes in neural activity. The devise placed to the scalp, TMS generates a magnetic field that stimulates underlying brain tissue, unsettling or enhancing cortical processing in localized areas ( Sliwinska, Vitilo & Devlin, 2014). This permits for precise manipulation of cognitive functions and provide insights into the timing and role of neural processes in real-time decision-making. In addition, consumer research, TMS provides a powerful technique to explore how the brain responds to marketing stimuli. By selectively activating or inhibiting regions associated with emotion, attention, and decision-making, researchers can examine how these changes affect purchasing attitudes and behavior. Recent studies using TMS on the dorsolateral prefrontal cortex (DLPFC) have shown that modulating this region can significantly alter consumer assessment and risk preferences during purchasing decisions ( Gupta et al., 2021). Moreover, this technique has been effectively used to study the influence of affective priming on product judgments, indicating that temporary disruption of emotion-processing circuits can modulate brand attitudes and consumer trust ( Zhang & Li, 2022). Research findings support the growing role of TMS in neuromarketing, where it helps decode, the neural mechanisms underlying consumer attention, preference formation, and decision-making, enabling marketers to develop empirically informed engagement strategies.

Indeed, TMS can directly influence consumption-related behaviours, such as delaying gratification or shifting preference toward serviceable versus hedonic products ( Ramsoy et al., 2021). Furthermore, emerging TMS applications in neuromarketing explore the modulation of empathy and social cognition through ethical consumption decisions, revealing that stimulating the right temporoparietal junction can alter perceived brand authenticity and trust ( Cian et al., 2022). Through effectively use these techniques is not merely a validation tool for neural correlates but a mechanism for probing and reshaping the cognitive architecture of consumer thought itself. This opens promising avenues for designing interventions in areas such as compulsive buying, financial risk behavior, and ethical advertising.

## Steady State Topography

Steady-State Topography (SST), introduced by Richard Silberstein in 1990, is a cutting-edge neurophysiological method for mapping neural activity in response to external stimuli. Initially developed for cognitive neuroscience, this device supports significant applications in neuromarketing and consumer neuroscience, especially in evaluating consumer perception, emotive engagement, and brand communication ( Maran, Moonisha, Patel, Mari Muthu, & Anbazhagan, 2021). In SST experiments, participants are exposed to audiovisual stimuli while a dim sinusoidal flicker is presented in their peripheral vision, causing a steady-state visually evoked potential (SSVEP). This neural response is measured across cortical areas to assess variations in cognitive and emotional processing. The specific concern of SST, particularly in neuromarketing, is its sensitivity to changes in latency, the delay between stimulus and neural response, which reflects long-term memory encoding, sustained attention, and emotional intensity ( Silberstein & Nield, 2021). By analysing latency shifts and cortical activation patterns over time, SST enables a more nuanced understanding of how consumers perceive advertising messages, process branding cues, and form durable attitudes toward products. Steady-State Topography-based new research demonstrated its ability to predict advertising effectiveness and product recall with high precision, making it an advanced tool for capturing subconscious consumer behaviour in real-world media environments.

## Magnetoencephalography

Magnetoencephalography (MEG) is a technique that measures brain activity by detecting potential magnetic fields on the scalp using sensitive detectors housed in a helmet placed on the head. Unlike other methods, MEG is not influenced by tissue type, such as blood, brain matter, or bone, and can provide spatial and temporal resolution to determine the depth of a point in the brain. The MEGs are capable of monitoring population neuronal activity in the brain, but the costs associated with their use are significant due to the requirement for a magnetic field-free environment. This device records electrical activity in the brain, offering high temporal resolution to trace the consumers’ responses to marketing stimuli in real time.

The Magnetoencephalography offer unique advantage in neuromarketing method through providing millisecond-level temporal resolution and superior spatial localization of brain activity compared to Electroencephalography, while remaining non-invasive unlike functional magnetic Resonance. This method is very magnificent to captures magnetic fields generated by neuronal activity which allowing precise tracking of consumer responses to marketing stimuli such as advertisements and product designs ( Lee et al., 2021; Morinaga et al., 2022; Dikker & Pineda, 2023). Undouble this unique method effectively maps cognitive processes like brand recall, emotional arousal, and decision-making pathways. This real-time, high-fidelity insight supports profound consumer profiling and enhances predictive modelling in marketing strategies.

## Current evidence and knowledge gaps in core consumer neuroscience constructs

The current state of consumer neuroscience, particularly through the lens of neuromarketing, sheds light on four fundamental constructs influencing consumer decision-making: attention, emotion, memory, and valuation. Despite significant advancements, each construct presents unique evidence alongside notable knowledge gaps and methodological constraints that temper the predictive power of these insights when applied singularly to real-world consumer behavior.

***Attention***

Neuromarketing leverages eye-tracking and electroencephalography (EEG) to provide granular data on consumers’ visual engagement and attentional focus toward marketing stimuli such as advertisements, product packaging, and digital content. These methods effectively capture moment-to-moment cognitive engagement, revealing how attentional resources are allocated during exposure to marketing material. However, attention primarily denotes an initial cognitive process, which while critical does not independently forecast purchase decisions or long-term brand loyalty. This limitation arises because attentional engagement measures lack the capacity to account for the complex, multifaceted determinants of actual consumer behavior beyond stimulus exposure ( Adhikari, 2023; Alsharif et al., 2021).

***Emotion***

Neurophysiological and peripheral psychophysiological tools such as EEG, facial electromyography, galvanic skin response, and heart rate variability have enriched the understanding of the emotional dimension of consumer interactions with brands and advertising. These approaches measure implicit affective responses that are often inaccessible through self-report. Nevertheless, emotional interpretation remains nuanced and complicated by individual differences including personal traits, cultural influences, and situational factors, which can modulate the valence and intensity of emotion elicited. Consequently, emotional response data although invaluable must be contextualized within these moderating variables to avoid misinterpretation or overgeneralization ( Alsharif et al., 2021, 2025; Dwivedi & Sharma, 2024).

***Memory***

Techniques such as functional magnetic resonance imaging (fMRI), EEG, and Steady-State Topography offer insights into the neural substrates of brand encoding, recognition, and recall processes. This neural-level understanding is vital for capturing how consumers process and store brand-related information. However, laboratory-based neuroimaging measures of memory activation do not consistently translate into long-term brand loyalty or predict actual purchasing behavior in the marketplace. This gap underscores a critical limitation: neural correlates of memory activation do not directly equate to behavioral outcomes influenced by diverse external and internal factors beyond memory alone ( Alsharif et al., 2021; Yao & Wang, 2024).

***Valuation***

Neuromarketing neuroimaging studies have made notable progress in mapping brain regions associated with reward processing, value assignment, and preference formulation during consumer choice tasks. These findings underscore the neural valuation signals as core cognitive components underpinning decision making. Yet, such valuation signals represent only part of a broader panorama; consumer purchase behaviors are additionally shaped by social contexts, cultural norms, economic considerations, and environmental influences, limiting the scope of prediction based solely on neural valuation ( Alsharif et al., 2021; Yao & Wang, 2024).

***Integration and methodological considerations***

The evidence collectively suggests that while neuromarketing methods offer rich, complementary insights into consumer cognition and affect, they should not be regarded as standalone indicators for predicting consumer behavior. Instead, neural data must be integrated with behavioral measures, consumer self-reports, and real-world sales or market outcomes to enhance the ecological validity and predictive accuracy of findings. Importantly, many neuromarketing studies face challenges including limited sample sizes, high costs, potential confounds, and issues related to reverse inference the problem of inferring specific mental states merely from observed neural activation patterns. Such limitations urge cautious interpretation and a multi-methodological approach that bridges neural data with longitudinal and contextually grounded behavioral research ( Gupta et al., 2025; Song et al., 2025; Yao & Wang, 2024).

### Emerging Directions and Knowledge Gaps

Future research should focus on:

Integrative frameworks are being developed to merge neural, behavioral, and contextual data throughout the entire consumer decision-making process, aiming to clarify how affective, cognitive, and motivational processes interact. Advanced computational methods, including machine learning and artificial intelligence, are utilized to interpret complex, multivariate neuromarketing data, enhancing the prediction of overall consumer behavior. Ethical issues in neuroscience-based marketing, such as privacy, data security, and informed consent, are being addressed to ensure the responsible use of consumer neural data. Longitudinal and field studies are being expanded to connect neural markers with real purchasing behaviors and sustained brand engagement, thus addressing the shortcomings of short-term laboratory assessments. Additionally, there is an exploration of less-studied areas like cross-modal interactions and diverse market segments to develop more universally applicable neuromarketing models ( Alsharif et al., 2025; Gupta et al., 2025; Yao & Wang, 2024).

The consumer neuroscience through neuromarketing has substantially advanced the comprehension of attention, emotion, memory, and valuation processes in consumer decision-making. However, the predictive power and practical application hinge upon appropriately integrating multimodal neural metrics with real-world behavioral indicators while carefully navigating limitations and ethical complexities inherent in this emerging field. Thus, the synthesis of current evidence indicates a promising yet nuanced landscape for neuromarketing, highlighting both its transformative potential and the critical need for sophisticated, ethically guided research designs moving forward ( Alsharif et al., 2021, 2025; Dwivedi & Sharma, 2024; Yao & Wang, 2024).

## Conclusion

Neuromarketing is most valuable when used as a complement to established behavioral and self-report methods, rather than as a tool for directly ‘reading’ consumers’ minds. By integrating neurophysiological insights with traditional marketing research, neuromarketing provides a deeper understanding of unconscious consumer responses, such as emotional engagement, attention, and memory encoding, which are often inaccessible through conventional methods alone. Techniques like EEG, fMRI, and TMS offer real-time, objective data that can refine marketing strategies, enhancing the precision of advertisements, pricing, and product designs. However, while these techniques have proven effective in capturing the intricacies of consumer behavior, they also face notable challenges. These include limitations related to spatial and temporal resolution, as well as ethical concerns surrounding privacy, consent, and potential manipulation. The future of neuromarketing depends on addressing these issues through the development of multimodal, longitudinal, and cross-cultural research, which can provide a more holistic view of consumer behavior. Furthermore, aligning neuromarketing with ethical standards and marketing theory will be crucial in ensuring its responsible and sustainable application. Ultimately, the long-term success of neuromarketing hinges on balancing scientific rigor with ethical responsibility, offering marketers not just deeper insights into consumer behavior, but also a path to more effective and ethically sound marketing practices.

## Data Availability

All data are publicly available and cited in the manuscript. No new data were generated, and no DOI applies.
