# Supplementary material for: The Neuroscience of Marketing: Noninvasive Neuromarketing Methods for Understanding Consumer Choice and Priorities
Source: F1000Res. 2026 Aug 29;14:1132. Originally published 2025 Oct 17. [Version 4] doi: 10.12688/f1000research.168220.4 (PMC13579028; doi:10.12688/f1000research.168220.4)
Supplement: Peer review history

# Reviewer report: Reviewer response for version 4

**DOI:** 10.5256/f1000research.207864.r516873

**Published:** 2026-09-15

**Authors:** Rupali Gill

**Affiliations:** 1 Chitkara University, Rajpura, India

1. Clarify the definition and classification of “non-invasive” methods, particularly PET.
2. Clearly identify the review type and describe the literature-search methodology.
3. Strengthen the method-selection matrix using concrete marketing applications.
4. Expand discussion of reverse inference, predictive validity, and ecological validity.
5. Add a practical ethical governance checklist/table.
6. Clarify the methodological role of TMS.
7. Perform comprehensive language editing and remove repetition.
8. Check technical terminology, frequency bands, figures/tables, and references.

Is the review written in accessible language?

Partly

Are all factual statements correct and adequately supported by citations?

Partly

Are the conclusions drawn appropriate in the context of the current research literature?

Yes

Is the topic of the review discussed comprehensively in the context of the current literature?

Yes

Reviewer Expertise:

Neuromarketing

I confirm that I have read this submission and believe that I have an appropriate level of expertise to confirm that it is of an acceptable scientific standard, however I have significant reservations, as outlined above.

# Reviewer report: Reviewer response for version 4

**DOI:** 10.5256/f1000research.207864.r515812

**Published:** 2026-09-14

**Authors:** Ramachandran K K

**Affiliations:** 1 GRD Institute of Management, Coimbatore, India

No futher comments. Approved.

Is the review written in accessible language?

Partly

Are all factual statements correct and adequately supported by citations?

Yes

Are the conclusions drawn appropriate in the context of the current research literature?

Partly

Is the topic of the review discussed comprehensively in the context of the current literature?

Yes

Reviewer Expertise:

Machine Learning,Artificial Intelligence,Learning Algorithms,Business,Deep Learning,Use Of Artificial Intelligence,Artificial Intelligence Technology,Consumer Behavior,Integration Of Artificial Intelligence,Internet Of Things,Artificial Intelligence Systems,Big Data

I confirm that I have read this submission and believe that I have an appropriate level of expertise to confirm that it is of an acceptable scientific standard.

## References

[1] : Augmented and Virtual Reality (AR/VR) in Marketing: Developing Immersive Client Experiences to Increase Engagement. *https://ieeexplore.ieee.org/document/10434889* .

[2] : Examining the Integration of Artificial Intelligence and Marketing Management to Transform Consumer Engagement. *https://ieeexplore.ieee.org/document/10545209/authors#authors* .

[3] : Utilising AI to Transform Marketing Management and Enhance Consumer Interaction Strategies. *https://ieeexplore.ieee.org/document/10545081* .

# Reviewer report: Reviewer response for version 4

**DOI:** 10.5256/f1000research.207864.r515815

**Published:** 2026-09-10

**Authors:** Singgih Santoso

**Affiliations:** 1 Universitas Kristen Duta Wacana Yogyakarta, Yogyakarta, Indonesia

Thank you for the revisions made to the manuscript. I have reviewed Version 4 in relation to my previous report. Several of my earlier concerns have been satisfactorily addressed, particularly the clarification of the review methodology and the strengthened synthesis of the method-selection matrix and knowledge gaps.

 However, two issues remain and would benefit from further clarification.

 First, the conceptual consistency regarding the term “noninvasive” still requires attention. Although PET has been removed from the abstract list of noninvasive methods, PET continues to be discussed in the main text despite its use of radioactive tracers. The manuscript should explicitly clarify that PET is not a noninvasive technique and explain why it is retained in the discussion or alternatively revise the relevant section to maintain consistency with the stated scope of the article. A clearer typology of the methods would also help resolve this issue.

 Second, the discussion of methodological and ethical limitations has been improved substantially, including issues related to privacy, data security, informed consent, and the limitations of predicting real-world consumer behavior. However, the ethical governance discussion would benefit from a clearer practical statement or brief checklist covering informed consent, privacy, data use, data security, and the appropriate limits of neuroadaptive marketing.

 Overall, Version 4 represents a substantial improvement and addresses most of the concerns raised in my previous review.

 I recommend **Approved with Reservations**, with the expectation that these two remaining issues be clarified in the next revision.

Is the review written in accessible language?

Partly

Are all factual statements correct and adequately supported by citations?

Yes

Are the conclusions drawn appropriate in the context of the current research literature?

Partly

Is the topic of the review discussed comprehensively in the context of the current literature?

Partly

Reviewer Expertise:

Marketing

I confirm that I have read this submission and believe that I have an appropriate level of expertise to confirm that it is of an acceptable scientific standard, however I have significant reservations, as outlined above.

# Reviewer report: Reviewer response for version 3

**DOI:** 10.5256/f1000research.198451.r468284

**Published:** 2026-06-29

**Authors:** Singgih Santoso

**Affiliations:** 1 Universitas Kristen Duta Wacana Yogyakarta, Yogyakarta, Indonesia

Article makes use of non-invasive neuromarketing techniques to assess how consumers make purchasing decisions. Such technologies include EEG, fMRI, MEG, TMS, Steady-State Topography, eye-tracking and peripheral psychophysiology, and are related to the core constructs of attention, emotion, memory and valuation. And the article also includes a method-selection matrix which links common marketing-related questions (e.g. ad testing, packaging, pricing) with applicable techniques and describes the trade-offs involved for both cost and temporal/spatial resolution versus ecological validity.

**comments**

 First, the scope and method of the review are still not clearly described. I would recommend that the authors state explicitly that this is a narrative/scoping review and briefly describe how they identified the relevant literature (e.g., the main databases searched, the years covered, the keywords used, and the basic criteria for inclusion). This will provide clarity regarding what is meant by “comprehensive” coverage and will respond to earlier concerns about the transparency of the review process.

 Second, the method- selection matrix is a nice addition; however, the synthesis of the matrix can be improved. It would be helpful for readers if the authors provided some specific examples to help them understand why certain methods or combinations of methods are better suited for use than others (e.g., pre-testing TV ads, evaluating sustainable branding, optimizing pricing). In addition, it would be helpful for the authors to include a short subsection that summarizes what we know and don’t know for each of the four main constructs (i.e., attention, emotion, memory, and valuation) at the end of the conclusions to help ground the conclusions in evidence from the literature review.

 third, the lack of consistency in terms and concept relating to ‘non-invasive’ measures is still a major issue. Those who previously reviewed research in this area pointed out that PET is one example that has complicating factors. The author should clarify the use of the term “non-invasive” by developing a typology (metabolic imaging, electrophysiology, neuromodulation, peripheral psycho-physiological, and behavioural / implicit).

 Fourth, a welcome addition to the paper is the inclusion of discussion about methodical and ethical challenges; although they were not explored in detail. The paper could benefit from a more rigorous examination of the issues raised by breaking down the issues using examples of situations where neuromarketing outcomes could not predict reality or where low sample sizes/inverse inference have led to conclusions that have been overgrown. I also propose developing a checklist of ethical practices and principles for practitioners regarding consent, privacy, use of data, and limitations of neuroadaptive marketing and including them in a table in this article to increase the practical relevance of the article.

**Overall recommendation**

 After reviewing the article and its revisions, I recommend it as approved, but with reservations. After clarifying their review method, providing more integration around their method selection matrix, strengthening their critique of methodology and ethical considerations, and improving the clarity of the language used to describe these issues, this paper will provide an important addition to the literature.

 NOTE:

 The Turnitin test shows that the article has a similarity score of 97% and an AI writing score of 57%, which proves that the article was generated by AI and contains plagiarism. In order for it to be published, please reduce the similarity score and ensure the AI writing score is BELOW 30%.

Is the review written in accessible language?

Partly

Are all factual statements correct and adequately supported by citations?

Yes

Are the conclusions drawn appropriate in the context of the current research literature?

Partly

Is the topic of the review discussed comprehensively in the context of the current literature?

Partly

Reviewer Expertise:

Marketing

I confirm that I have read this submission and believe that I have an appropriate level of expertise to confirm that it is of an acceptable scientific standard, however I have significant reservations, as outlined above.

# Reviewer report: Reviewer response for version 2

**DOI:** 10.5256/f1000research.195165.r468285

**Published:** 2026-03-30

**Authors:** Ramachandran K K

**Affiliations:** 1 GRD Institute of Management, Coimbatore, India

The manuscript presents a narrative review of non-invasive neuromarketing methods employed to examine consumer behavior. It discusses key techniques such as EEG, fMRI, MEG, TMS, eye-tracking, and Steady-State Topography, and connects them to core constructs including attention, emotion, memory, and valuation. The authors seek to move beyond purely descriptive accounts by providing a more comparative and integrative framework that incorporates a method-selection matrix and an analysis of trade-offs among cost, ecological validity, and temporal versus spatial resolution. In addition, it outlines potential directions for future research, particularly emphasizing the importance of multimodal approaches and more ecologically valid study designs.

**Strength:**

1. The paper successfully links neuroscientific methods with core marketing constructs such as attention, emotion, memory, and valuation.
2. It incorporates recent literature, reflecting current developments in the field.
3. A well-written article that is appropriate to the journal.

**Weakness:**

1. The readability is partially effective as several sections contain errors in grammar and phrasing and are frequently dense and repetitive. This makes it difficult to follow the arguments.
2. Ensure consistency

**Comments:**

 The manuscript addresses a relevant and emerging topic and offers a valuable integrative perspective on non-invasive neuromarketing methods. The effort to connect techniques with core consumer behavior constructs and to propose a comparative framework is a notable strength **. **To avoid redundancy, simplify the sentence structure to make it easier to follow.

 The manuscript presents a meaningful contribution to the field and is suitable for indexing with a moderate revision addressing clarity, and consistency.

Is the review written in accessible language?

Partly

Are all factual statements correct and adequately supported by citations?

Yes

Are the conclusions drawn appropriate in the context of the current research literature?

Partly

Is the topic of the review discussed comprehensively in the context of the current literature?

Yes

Reviewer Expertise:

Marketing, Management, AI, Retail Management

I confirm that I have read this submission and believe that I have an appropriate level of expertise to confirm that it is of an acceptable scientific standard, however I have significant reservations, as outlined above.

# Reviewer report: Reviewer response for version 1

**DOI:** 10.5256/f1000research.185388.r432458

**Published:** 2025-12-29

**Authors:** Fitri Aprilianty

**Affiliations:** 1 Yamaguchi University, Yamaguchi, Japan; 2 Institut Teknologi Bandung Sekolah Bisnis Manajemen (Ringgold ID: 479250), Bandung, West Java, Indonesia

**1.** Is the topic of the review discussed comprehensively in the context of the current literature? ( **Partly)**

 The paper covers many key tools and cites a broad range of literature, including recent work on EEG, fMRI, MEG, TMS, eye-tracking, and neuromarketing ethics.

 However, the discussion is mostly descriptive and technique-by-technique, with limited synthesis across studies, and there is no explicit method for selecting or delimiting the reviewed literature. Important dimensions such as cross-modal comparisons, application domains (e.g. branding, pricing, digital platforms), and known controversies (e.g. reverse inference, effect sizes, replicability) are touched on only briefly, if at all.

- Clarify the **scope and method by **state explicitly that this is a narrative/scoping review rather than a systematic review, and briefly describe how the authors identified and selected the literature 
- Add at least one **integrative section or figure** (e.g. a table or matrix) that:
  - compares techniques on temporal/spatial resolution, cost, ecological validity, and typical marketing questions they address; and
  - summarises “what we know” after two decades of consumer neuroscience (e.g. robust findings on value coding, attention, memory, and predictive validity for real-world outcomes).
- Expand the **“big picture”** integration in the conclusion: what are the main areas of consensus/disagreement in the literature, and where are the most promising gaps (e.g. multi-modal approaches, longitudinal designs, cross-cultural neuromarketing)?

2. Are all factual statements correct and adequately supported by citations?

 Non-invasive” and invasive inconsistency. The title and abstract frame the review as focusing on *non-invasive* methods, listing PET alongside fMRI, EEG, etc. Later, the PET section correctly notes that PET involves injection of radioactive tracers and is therefore invasive and less commonly used. This is **internally inconsistent** and conceptually misleading. Either (a) remove PET from the “non-invasive” framing in the title/abstract and clearly state that PET is *minimally invasive* and now rarely used in consumer work, or (b) broaden the title/scope beyond “non-invasive” and clearly distinguish invasive vs non-invasive methods throughout. 

 The paper sometimes groups very different tools under “non-invasive neuromarketing techniques” (e.g. PET, TMS, implicit association tests, biometric sensors) without clearly distinguishing neuroimaging / electrophysiology from behavioural / psychophysiological measures.

 Use a consistent typology (e.g. *metabolic imaging* (fMRI, PET), *electrophysiological* (EEG, MEG, SST), *neuromodulation* (TMS), *peripheral psychophysiology* (GSR, HR, facial EMG), *behavioural implicit measures*).

 4. Are the conclusions drawn appropriate in the context of the current research literature? 

 However, the conclusions are quite general and do not always flow directly from the preceding sections. They would be more impactful if they more clearly summarised what the reviewed evidence actually shows and what remains uncertain.

 Anchor conclusions to specific evidence. For example, summarise key robust findings (e.g. value coding in vmPFC/NAcc, predictive power of certain neural markers for campaign success, reliability of frontal alpha asymmetry for approach–avoidance tendencies).

 Differentiate short-term promise vs long-term challenges

Is the review written in accessible language?

Yes

Are all factual statements correct and adequately supported by citations?

Partly

Are the conclusions drawn appropriate in the context of the current research literature?

Partly

Is the topic of the review discussed comprehensively in the context of the current literature?

Partly

Reviewer Expertise:

Consumer Behavior, marketing, neuromarketing

I confirm that I have read this submission and believe that I have an appropriate level of expertise to state that I do not consider it to be of an acceptable scientific standard, for reasons outlined above.

# Reviewer report: Reviewer response for version 1

**DOI:** 10.5256/f1000research.185388.r425175

**Published:** 2025-12-11

**Authors:** Jesús Jaime Moreno Escobar

**Affiliations:** 1 ESIME - Escuela Superior de Ingeniería Mecánica y Eléctrica Unidad Zacatenco, Mexico City, Mexico

The proposal advocates for the use of non-invasive neuromarketing techniques—such as fMRI, EEG, PET, MEG, and eye-tracking—to understand the subconscious drivers of consumer behavior. This approach aims to overcome the limitations of traditional methods like surveys and focus groups by directly measuring brain activity and physiological responses.

**Advantages:**

1. **Access to Subconscious Processes:** The primary advantage is the ability to bypass the limitations of self-reporting. These techniques can reveal unconscious emotional, cognitive, and decision-making processes that consumers cannot or will not articulate, providing more authentic insights.
2. **Objective and Rich Data:** Neuroimaging provides objective, biological data on consumer responses to marketing stimuli (ads, products, brands). This allows for a deeper understanding of the neural mechanisms behind attention, emotional engagement, memory encoding, and preference formation.
3. **Improved Predictive Power:** Studies show that neural data can predict market outcomes, such as advertising success and product desirability, more accurately than traditional methods. This allows companies to refine product designs, pricing, and promotional content with greater confidence, potentially reducing market failure.
4. **Real-Time and High-Resolution Insights:** Techniques like EEG and MEG offer very high temporal resolution (milliseconds), allowing researchers to track consumer responses in real time. This is crucial for understanding the immediate impact of fast-paced marketing stimuli.
5. **Application to Innovation and Personalization:** Neuromarketing supports new product development by testing prototypes and can enable hyper-personalized, "neuroadaptive" marketing where content is dynamically tailored based on real-time neural feedback.

**Disadvantages:**

1. **High Cost and Complexity:** Many of these tools, particularly fMRI and MEG, are extremely expensive to acquire and operate. They also require highly specialized expertise in both neuroscience and data analysis, limiting their accessibility.
2. **Methodological Limitations:** Each technique has inherent trade-offs. fMRI has excellent spatial resolution but poor temporal resolution and a high cost. EEG is portable and has great temporal resolution but poor spatial resolution and cannot probe deep brain structures.
3. **Ethical and Privacy Concerns:** The field raises significant ethical questions about "mind-reading," consumer manipulation, and data privacy. There is public apprehension about the potential for brands to exploit subconscious biases and vulnerabilities.
4. **Interpretation Challenges:** Linking specific neural signals to complex psychological constructs like "brand loyalty" or "purchase intent" is inferentially complex. The data requires careful interpretation, and standardized protocols are still evolving, which can lead to variability in results.
5. **Limited Adoption and Transparency:** The high barriers to entry have slowed widespread adoption in market research. Furthermore, businesses are often hesitant to disclose their use of neuromarketing, which fuels skepticism about its application.
  Dear Editor,
  Thank you for the opportunity to review this manuscript. I have evaluated it based on standard scholarly criteria. My overall recommendation is to **reject the manuscript in its current form, but with encouragement for a significant resubmission**. The manuscript addresses a topic of considerable interest but requires substantial revisions to meet the journal's standards. Below is my detailed evaluation.
**1. Originality / Novelty**
  The topic of applying non-invasive neuromarketing techniques is inherently relevant. However, the manuscript's originality is limited. It functions primarily as a descriptive catalog of established methods (fMRI, EEG, PET, MEG) and their general advantages and disadvantages, rather than presenting a novel synthesis, a new conceptual framework, or a critical gap analysis that would distinguish it from other existing reviews in the field. The authors have not clearly demonstrated how their approach or synthesis offers a new perspective compared to the extant literature.
**2. Significance of Content**
  The content is of significant potential interest to the fields of marketing, consumer behavior, and applied neuroscience. Understanding the subconscious drivers of consumer decision-making is a central challenge, and a well-executed review that critically appraises the value and limitations of these tools would be highly valuable. The potential for impact is present, but it is not fully realized in the current draft.
**3. Quality of Presentation**
  This is a major weakness of the manuscript. The authors **do not use a consistently readable level of English**. Grammatical errors, awkward phrasing, and unclear syntax are prevalent throughout the text, which significantly hinders comprehension and detracts from the scholarly tone. Furthermore, the logical structure is problematic; the justification for the review and the clear articulation of the main problem it aims to solve are not effectively exposed, making the manuscript's core contribution difficult to discern.
**4. Scientific Soundness**
  The scientific soundness is compromised by several factors. While the reference list is relevant, it is not sufficiently current, with only 45% of the 88 references from the last five years. This undermines the manuscript's claim to represent the state-of-the-art in a rapidly evolving field. More critically, the review lacks a balanced critical perspective. It lists disadvantages (e.g., cost, ethical concerns) but does not engage with them in a substantive way that would provide a rigorous appraisal of the field's validity and reliability. The manuscript describes rather than critiques.
**5. Interest to the Readers**
  A robust and well-written review on this topic would be of high interest to the diverse readership of *F1000Research*, including neuroscientists, marketing professionals, and behavioral economists. The subject matter aligns well with interdisciplinary research. However, the current presentation issues significantly reduce its accessibility and, therefore, its potential impact.
**6. Overall Merit and Critical Concern**
  The manuscript has a strong foundational premise and covers a valuable topic. However, its current overall merit is low due to the critical issues in presentation, originality, and scientific rigor. The **originality report shows a 19% similarity index, which is acceptable, but also flags that 71% of the content has a high probability of being AI-generated.** This is a severe concern. The stilted language, lack of a coherent narrative voice, and descriptive rather than critical analysis are consistent with this finding. For a scholarly publication, the intellectual synthesis and critical perspective must be demonstrably the product of the authors' expertise.
**Recommendation:**
  I recommend **rejecting the current manuscript but encouraging a resubmission** after the authors undertake a thorough, top-to-bottom revision. The authors should:
6. Completely rewrite the manuscript to ensure clarity, fluency, and a strong, critical authorial voice, decisively addressing the concerns of AI-assisted writing.
7. Restructure the paper to clearly state its unique contribution, define a specific gap in the literature, and provide a compelling justification for the review.
8. Update the literature review significantly to include more recent publications (post-2019).
9. Move beyond description to provide a critical, balanced analysis that deeply engages with both the advantages and the methodological and ethical challenges of neuromarketing.
10. The core topic has great potential, and I would be interested in reviewing a fundamentally revised version that demonstrates genuine scholarly engagement and critical thought.

Is the review written in accessible language?

Yes

Are all factual statements correct and adequately supported by citations?

Yes

Are the conclusions drawn appropriate in the context of the current research literature?

Yes

Is the topic of the review discussed comprehensively in the context of the current literature?

No

Reviewer Expertise:

COmputer Vision, Data Analitycs

I confirm that I have read this submission and believe that I have an appropriate level of expertise to state that I do not consider it to be of an acceptable scientific standard, for reasons outlined above.
